# Control of Angiogenesis via a VHL/miR-212/132 Axis

**DOI:** 10.3390/cells9041017

**Published:** 2020-04-19

**Authors:** Zhiyong Lei, Timothy D. Klasson, Maarten M. Brandt, Glenn van de Hoek, Ive Logister, Caroline Cheng, Pieter A. Doevendans, Joost P. G. Sluijter, Rachel H. Giles

**Affiliations:** 1 Department of Cardiology, Division Heart and Lungs, University Medical Center Utrecht, Heidelberglaan 100, 3584 CX Utrecht, The Netherlands; leizhiyong@gmail.com (Z.L.); P.Doevendans@umcutrecht.nl (P.A.D.); 2 Department of Nephrology & Hypertension, Division Internal Medicine, University Medical Center Utrecht, Heidelberglaan 100, 3584 CX Utrecht, The Netherlands; timklasson@gmail.com (T.D.K.); i.logister@hubrecht.eu (I.L.); K.L.Cheng-2@umcutrecht.nl (C.C.); 3 Experimental Cardiology, Department of Cardiology, Thoraxcenter, Erasmus MC, University Medical Center Rotterdam, 3015 GD Rotterdam, The Netherlands; m.brandt@erasmusmc.nl; 4 Department of Medical Genetics, University Medical Center Utrecht, Heidelberglaan 100, 3584 CX Utrecht, The Netherlands; G.vandeHoek@umcutrecht.nl; 5 Netherlands Heart Institute, Catharijnesingel 52, 3511 GC Utrecht, The Netherlands; 6 Central Military Hospital Utrecht, Lundlaan 1, 3584 EZ Utrecht, The Netherlands; 7 UMC Utrecht Regenerative Medicine Center, University Medical Center, 3584 CT Utrecht, The Netherlands

**Keywords:** VHL loss of function, microRNA-212/132, angiogenesis

## Abstract

A common feature of tumorigenesis is the upregulation of angiogenesis pathways in order to supply nutrients via the blood for the growing tumor. Understanding how cells promote angiogenesis and how to control these processes pharmaceutically are of great clinical interest. Clear cell renal cell carcinoma (ccRCC) is the most common form of sporadic and inherited kidney cancer which is associated with excess neovascularization. ccRCC is highly associated with biallelic mutations in the von Hippel–Lindau (VHL) tumor suppressor gene. Although upregulation of the miR-212/132 family and disturbed VHL signaling have both been linked with angiogenesis, no evidence of a possible connection between the two has yet been made. We show that miRNA-212/132 levels are increased after loss of functional pVHL, the protein product of the VHL gene, in vivo and in vitro. Furthermore, we show that blocking miRNA-212/132 with anti-miRs can significantly alleviate the excessive vascular branching phenotype characteristic of vhl^−/−^ mutant zebrafish. Moreover, using human umbilical vascular endothelial cells (HUVECs) and an endothelial cell/pericyte coculture system, we observed that VHL knockdown promotes endothelial cells neovascularization capacity in vitro, an effect which can be inhibited by anti-miR-212/132 treatment. Taken together, our results demonstrate an important role for miRNA-212/132 in angiogenesis induced by loss of VHL. Intriguingly, this also presents a possibility for the pharmaceutical manipulation of angiogenesis by modulating levels of MiR212/132.

## 1. Introduction

Clear cell renal cell carcinoma (ccRCC), the most common form of sporadic and inherited kidney cancer, is highly associated with mutations in the von Hippel-Lindau (*VHL*) gene [1,2]. The protein product of the *VHL* gene (pVHL) is an E3 ubiquitin ligase involved in the degradation of hypoxia-inducible transcription factor subunits (HIF1α). Under normal oxygen tension, hydroxylated HIF1α can be recognized by the ubiquitin ligase complex containing pVHL and rapidly degraded. Upon hypoxia or loss of functional pVHL, HIF1α-subunits can no longer be hydroxylated and begin to accumulate. Stabilized HIF1α activates the expression of a large suite of downstream target genes (Erythropoietin (*EPO)*, vascular endothelial growth factor (*VEGF*), etc), the actions of which are vital to promote angiogenesis. However, many of the changes initiated by the stabilization of HIF1α, such as increased angiogenesis, an upregulation of antiapoptotic signaling, and a shift to anaerobic glycolosis, can contribute to tumor growth and survival. People born with a mutation in one *VHL* allele may acquire somatic mutations in the second allele, resulting in consequent angiogenic symptoms and a variety of tumors, including ccRCC [3]. Another hallmark of ccRCC is the activated phosphatidylinositol-4,5-bisphosphate 3-kinase (PI3k)/AKT pathway signaling, higher levels of which is significantly correlated with a worse survival rate [2], although the mechanism by which this occurs is still not fully understood.

MicroRNAs (miRNAs) are small noncoding RNAs that posttranscriptionally regulate the expression of groups of target genes by inhibition of the translation of their targeting messenger RNAs (mRNAs) or marking these mRNAs for degradation. miRNAs are key regulators in many physiological and pathological processes [4], including the dynamic regulation of ccRCC during tumor progression [2]. By promoting the expression of vascular endothelial growth factor (*VEGF*), VHL/HIF1 signaling increases Cyclic adenosine monophosphate (cAMP) response element binding protein (CREB) levels, a transcription factor which upregulates the expression of pro-angiogenic miR-212/132 [5]. This implies that pVHL loss-of-function would stimulate miR-212/132 expression and therefore contribute to excessive angiogenesis. In this study, using a combination of cellular models, patient ccRCC material with biallelic loss of VHL and a previously described vhl^−/−^ mutant zebrafish model, we show that miR-212/132 is upregulated after VHL knockdown or mutation and that this upregulation is at least partially responsible for pro-angiogenic effects. In order to grow, cancer tissues such as ccRCC have developed various strategies to provide sufficient blood supply by promoting angiogenesis [6]. Many of the common pharmaceutical treatments for ccRCC, such as sunitinib, use the strategy of reducing pathophysiological angiogenesis [7]. Conversely, many different strategies have been tested to improve perfusion of certain ischemic tissues or engineered tissue constructs by promoting neovascularization, which is essential for functional recovery of the organ after ischemic events or survival of transplanted engineered tissue constructs [8]. A scarcity of functional pVHL induces excessive vascular outgrowth, which further is enhanced by miR-212/132 expression, providing an exciting target for the modulation of angiogenesis.

## 2. Materials and Methods

### 2.1. MicroRNA In Situ Hybridization

microRNA in situ hybridization was performed with a modified microRNA in situ hybridization method as described [9]. Formalin-fixed paraffin-embedded tumor tissue from two ccRCCs and one healthy donor kidney dating from September 2006 were collected from the pathology archives of the University Medical Centre Utrecht (UMCU) after authorization of the UMCU institutional review board in accordance with Dutch medical ethical guidelines. Sequencing results of *VHL* identified no variants in the normal healthy kidney. However, in ccRCC #1, in addition to the already known germline deletion of *VHL* exons 1 and 2, an additional somatic mutation was found in the tumor (c.277delG/p.Gly93Ala_fs_x158). ccRCC #2 has a germline mutation c.266T> p.Leu89Pro and a somatic mutation of c.419-420delTC/p.Leu140Gln_fs_x142. Mutation analysis of these tumors has been previously published [10].

Paraffin samples were first deparaffinized with tissue clear (Cat# 1426, SAKURA) followed with 10 min of proteinase K treatment (5μg/ml, Cat# 03115828001, Roche). Hybridization was performed with 10 nM DIG-labeled miRCURY LNA miRNA detection probes in hybridization buffer (Urea (2 M), 2.5× SSC, 1× Denhardt’s, 200 µg/ml yeast tRNA, 0.1% CHAPS, 0.1% Tween, and 50mg/ml heparin) for miR-132 (Cat# 38031-15, Exiqon). Sections were subsequently incubated with anti-DIG alkaline phosphatase antibody (1:1,500, Cat# 1093274, Roche). To block endogenous alkaline phosphatase activity, sections were incubated with levamisole solution (Cat. X3021, DAKO), followed by NBT/BCIP (Cat# K0598 DAKO) incubation for visualization. A light Eosin counter staining was performed to visualize histology of the tissue. Images were taken with an Olympus microscope (BX53) under bright field.

### 2.2. Cell Culture and Transfection

Human umbilical vascular endothelial cells (HUVECs) were cultured in EGM2 (Lonza, cat# cc-3156) according to manufacturer’s instructions, and all experiments were performed before passage 7. HUVECS were either transfected with validated siVHL (ID: s14790), siPTEN (ID: s61222), silencer select negative control #1 (cat# 4390843), mirVana miRNA mimic negative control (cat# 4464085), hsa-miR-132-3p mimics (ID: MC10166), hsa-miR-212-3p mimics (ID: MC10340), mirVana miRNA inhibitor control1 (cat# 4464077), hsa-miR-132-3p inhibitor (ID: AM10166), or hsa-miR-212-3p inhibitor (ID: AM10340) (all from Life Technologies) using Lipofectamine 2000 (Life Technologies). The transfection was performed with a final concentration of 20 nM in opti-MEM reduced-serum medium (Cat# 31985062, Life Technologies) and replaced with fresh EGM2 after 6 hours. Cells were harvested 72 hours after transfection for protein or RNA analysis.

### 2.3. RNA Isolation and RT-PCR

Total RNA was isolated with Tripure Isolation Reagent following manufactory’s instructions (Roche Applied Science) and treated with Dnase to remove potential genome DNA contaminations. cDNA was synthesized using the iScript^TM^ cDNA Synthesis Kit (Bio-Rad). Quantitative real-time polymerase chain reaction (qRT-PCR) was performed with iQ SYBR Green Supermix (Bio-Rad). The following primers were used for detection of human genes: *GAPDH* forward 5′-GGCATGGACTGTGGTCATGA-3′ and reverse 5′-TTCACCACCATGGAGAAGGC-3′; *PTEN* forward 5′-TGGATTCGACTTAGACTTGACCT-3′ and reverse 5′-GGTGGGTTATGGTCTTCAAAAGG-3′; *VHL* forward 5′-CAGCTACCGAGGTCACCTTT-3′ and reverse 5′-CCGTCAACATTGAGAGATGG-3′; the following primers are used for *zebrafish*: *ptena* forward 5′-CCAGCCAGCGCAGGTATGTGTA-3′ and reverse 5′-GCGGCTGAGGAAACTCGAAGATC-3′; *ptenb* forward 5′-GCTACCTTCTGAGGAATAAGCTGG-3′ and reverse 5′-CTTGATGTCCCCACACACAGGC-3′; *rpl13α* forward 5′-TCTGGAGGACTGTAAGAGGTATGC-3′ and reverse 5′-AGACGCACAATCTTGAGAGCAG-3′ (All primers from Integrated DNA Technologies, Coralville, Iowa, USA).

### 2.4. In Vitro Angiogenesis Assay

HUVECs (Lonza) and human brain vascular pericytes (Cat#1200, Sciencell) were cultured on gelatin-coated plates in EGM2 medium (Lonza cat# cc-3156) and DMEM (10% FCS; Lonza), respectively, in 5% CO_2_ at 37 °C. Lentiviral transduced HUVECs expressing green fluorescent protein (GFP) and pericytes expressing red fluorescent protein (RFP) were used between passage 6–8. HUVECs were transfected either with siRNA or anti-miRs as described above. In order to monitor the effects of miR-132 and miR-212 in angiogenesis, transfected HUVEC-GFP and Pericytes-dsRed were suspended in a 2.5 mg/ml collagen type I (BD Biosciences) as described by Stratman [11]. Cocultures were imaged after 48 h and 120 h incubation in 5% CO_2_ at 37 °C by fluorescence microscopy, followed by automated thresholding and skeletonization of the images using a commercial analysis system (Angiosys, Buckingham, UK). These images were used for automated tubule length measurement, junction measurement, and other analyses according to manufacturer’s instructions.

### 2.5. Zebrafish

Experiments were conducted in accordance with Dutch guidelines for the care and use of laboratory animals, with the approval of the Animal Experimentation Committee (DEC) of the Royal Netherlands Academy of Arts and Sciences (KNAW). Mutant *zebrafish* possessed the previously described vhl^hu2117^ mutation [12]. For RNA collection, *zebrafish* embryos were collected at 5 dpf based on these phenotypes and RNA was collected using Trizol reagent (Ambion) as per manufacturer’s instructions.

### 2.6. Injections and Visualizations

Wild-type and mutant *Zebrafish* embryos were injected at the 1–2-cell stage with 1 nL of 5 uM of the same miRNA mimics and antagonists used for the cell culture experiments described above. Mimics and antagonists were diluted in pure water with 0.1% phenol red for visualization and injected using a nanoject2000 microinjector (World Precision Instruments). *Zebrafish* were selected without bias at 5dpf and then imaged using an LSM700 microscope (Zeiss). DNA was then collected from theses embryos using lysis buffer, and then embryos were genotyped using a KASP™ genotyping system (LGC Genomics, Teddington, Middlesex, England) kit designed against the *vhl^hu2117^* mutation or by sanger sequencing using the following primers: Fw: 5’-TAA GGG CTT AGC GCA TGT TC-3’ and Rv: 5’-CGA GTT AAA CGC GTA GAT AG-3’.

### 2.7. Statistical Analysis

Data was analyzed with Graphpad Prism 6 and comparisons were performed with student t-test or paired *t*-test between two groups and with ANOVA for more than two groups. Data are presented as mean ± SEM. *p*-values are indicated as follows: * *p* < 0.05; ** *p* < 0.01; *** *p* < 0.001; *p* < 0.05 is considered significant.

## 3. Results

**Figure 1:**
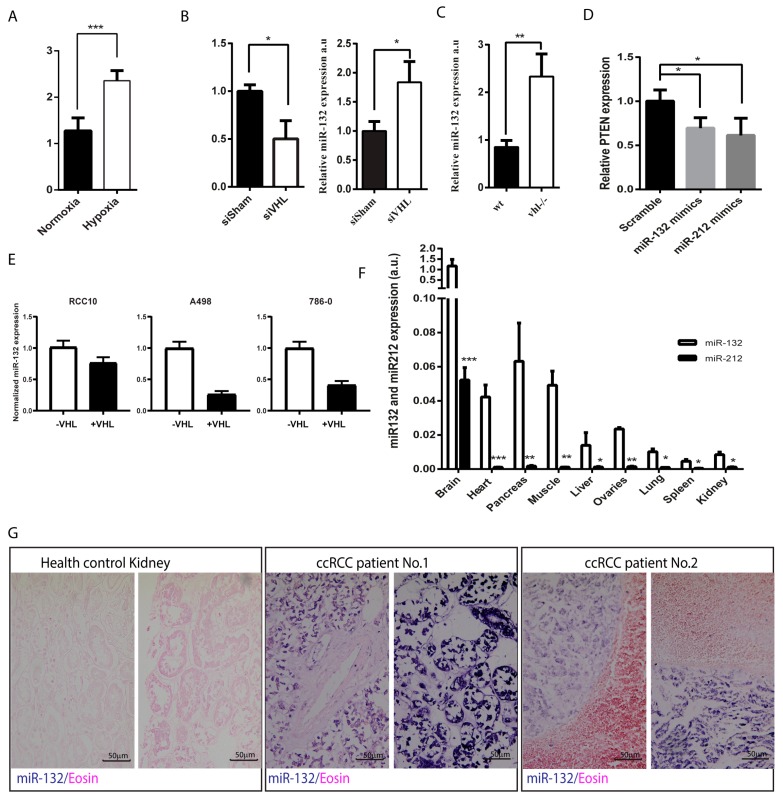
Characterization of miR-132 expression under hypoxic and pseudo-hypoxic conditions: (**A**) The expression of miR-132 in human umbilical vascular endothelial cells (HUVECs) under normoxia and hypoxia as compared by qPCR. (**B**) The expression of von Hippel–Lindau (VHL) in HUVECs after transfection with siRNA against VHL and the expression of miR-132 in siSham and siVHL transfected HUVECs as compared by qPCR. (**C**) The expression of miR-132 in wildtype (WT) and vhl^−/−^ mutant zebrafish as compared by qPCR. (**D**) The expression of known miR-132 target PTEN (phosphatidylinositol-3,4,5-trisphosphate 3-phosphatase) in HUVECs treated with miR-132/212 mimics versus control as compared by qPCR. (**E**) The expression of miR-132 in established VHL^−/−^ lines RCC10, A498, and 786-0 as well as the same lines reconstituted with ectopic VHL. The presented data is a mean of 3 in-depended PCR experiments with counting error. (**F**) Relative expression of miR-132 and 212 in different tissues in mouse. Note miR-132 expression is considerably higher than miR-212. *n* = 3. (**G**) The expression of miR-132 in healthy kidney tissue and ccRCC from two patients with known bilateral VHL mutations in their tumor as shown by miR-132 in situ hybridization. miR-132 in situ is in purple blue. Light eosin counterstaining appears pink. * *p* < 0.05; ** *p* < 0.01; *** *p* < 0.001

miRNA-212/132 are transcribed as a single RNA transcript and subsequently processed into two different mature microRNA miR-132 and miR-212. Due to the different efficiency in pre-miR processing, miR-132 is the dominant family member, as shown in Figure 1F. In most of the tissues, the expression of miR-212 are hardly detectable with the only exception being the brain. For this reason, mir-132 was used as the primary target in this study. We first examined the expression of miR-132 in relation to VHL loss-of-function and (pseudo)-hypoxic signaling. We found that endothelial cells grown in hypoxic conditions display significantly elevated levels of miR-132 expression (Figure 1A). We observe similar effects in HUVECs transfected with siRNA targeting *VHL* mRNA relative to those treated with non-targeting siRNA (Figure 1B). miR-212/132 are well conserved in most species, including zebrafish (Supplementary Figure S1A). To confirm this effect in vivo, we used a previously established zebrafish model of *VHL* deficiency [12,13,14]. Like our cell models, *vhl*^−/−^ zebrafish also show an increased level of expression of miR-132 (Figure 1C). In isogenic cell lines taken from human ccRCC *VHL*^−/−^ tumors, the expression of miR-132 is reduced upon *VHL* reconstitution with ectopic *VHL* (Figure 1E). The reduction of miR-132 expression is present in all cell lines upon VHL introduction but less pronounced in the RCC10, which might be related to a cell-line-specific genetic alteration on top of the VHL disruption that affects the miR-132 pathway. To assess the functional consequences of miR-212/132 loss in these cells, we examined the expression of a known target mRNA of this miRNA family. PTEN, phosphatidylinositol-3,4,5-trisphosphate 3-phosphatase, antagonizes the activity of phosphatidylinositol-4,5-bisphosphate 3-kinase (PI3K), suppressing cellular proliferation, cell survival, and angiogenesis by inactivating the PI3K-driven AKT signaling pathway [15]. *PTEN* has been predicted to be a potential target of miR-212/132 in humans by targetscan (Supplemental Figure S1B), and the rat homologue of *PTEN* has been shown to be targeted by miR-212/132 in rat vascular smooth muscle cells [16]. Moreover, downregulation of PTEN has been significantly correlated with lower survival rate in ccRCC patients [2]. We reasoned that upregulated miR-212/132 upon mutation or silencing of *VHL* could result in the subsequent reduction of PTEN. We therefore examined PTEN expression in HUVEC cells transfected with miR-212/132 mimics and found that the expression of PTEN was significantly reduced in these cells (Figure 1D). In order to assess the relative effects of miR-212 versus miR-132, we examined the differential expression of these miRNA in different mouse tissues. miR-212 is found at extremely low levels in the tissues tested, except brain tissue (Figure 1F). Based on this information, we examined miR-132 expression in histology slides taken from ccRCC tumors using microRNA in situ hybridization. In agreement with our previous qPCR results, we observed widespread overexpression of miR-132 in tumor material from ccRCC samples with biallelic *VHL* mutations proven by sequencing (Figure 1E). These results demonstrate that miR-132 is increased in response to the pseudo-hypoxia induced by the lack of functional pVHL, which eventually leads to overexpression of miR-132.

**Figure 2:**
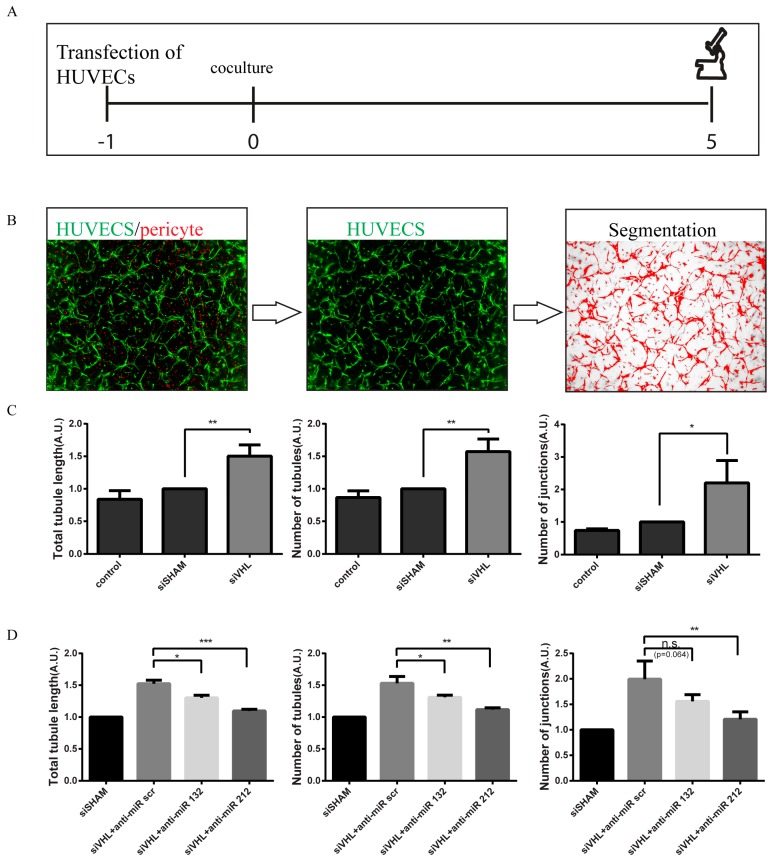
Reduced levels of VHL enhances endothelial cell neovascularization capacity and can be inhibited by blocking miR-132 or miR-212. (**A**) Schematic outline of the coculture experiment with HUVECs and pericytes. (**B**) Representative images showing the analysis process of tubular structures in the endothelial cells and pericytes coculture assay. (**C**) VHL siRNA knockdown in HUVECs enhances endothelial cell neovascularization capacity. (**D**) Blocking miR-132/212 inhibits neovascularization enhancement induced by VHL knockdown. Cell images are used to produce skeletonized 2D images which can be analyzed automatically. * *p* < 0.05; ** *p* < 0.01; *** *p* < 0.001

To assess the functional consequences of miR-212/132 expression in a *VHL*-null environment, we used an in vitro coculture assay designed to gauge angiogenesis. In agreement with the important role of VHL in HIF1 degradation, knockdown of VHL in HUVEC/pericyte coculture shows significantly more vascular junctions, tubule number, and total tubular length as compared to siSham control treatment (Figure 2A–C). To evaluate whether the pro-angiogenic effects of *VHL* silencing are mediated by a downstream increase in miR-212/132, GFP-labelled HUVECs were treated with anti-miRs against miR-212/132 in combination with siRNA targeting *VHL*. Inhibiting the action of miR-212/132 reduced the excessive angiogenetic response induced by the silencing of *VHL* significantly (Figure 2D), suggesting that VHL-regulated angiogenesis is at least partially mediated by the upregulation of miR-212/132.

**Figure 3:**
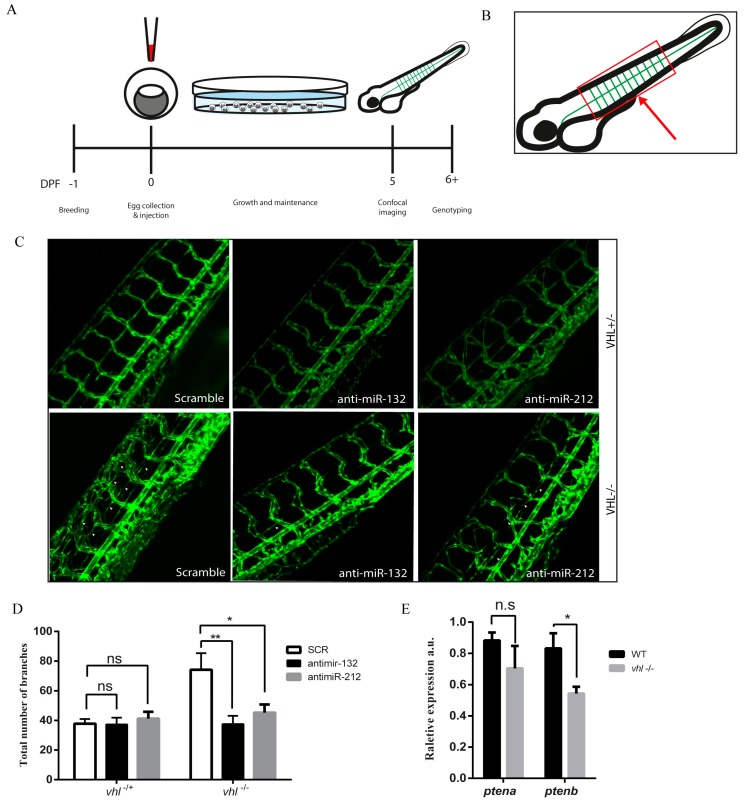
Inhibition of miR-132 or miR-212 suppresses VHL loss of function-induced vasculature outgrowth in zebrafish. (**A**) Schematic outline of the zebrafish embryo microinjection experiment. microRNA mimics and anti-miRs are injected into the yolk of the eggs on day 0 and imaged with a confocal microscope on day 5. (**B**) Schematic cartoon showing the area of the zebrafish embryo that is imaged after microinjection. The cloaca is marked with a red arrow. The imaging area is shown with a red box. The vessels of the tail are shown in green. (**C**) Representative images of zebrafish tail vascular structures in vhl^+/−^ and vhl^−/−^ zebrafish after injection with scrambled or miR-132 and miR-212 inhibitors. White arrows designate examples of structures which have been scored as branches. (**D**) Quantification of vascular branching in zebrafish tail structures after injection with scrambled control inhibitors, miR-132 inhibitors, or miR-212 inhibitors. (**E**) The expression levels of ptena and ptenb in WT and vhl^−/−^ zebrafish determined by qPCR. * *p* < 0.05; ** *p* < 0.01.

**Figure 4:**
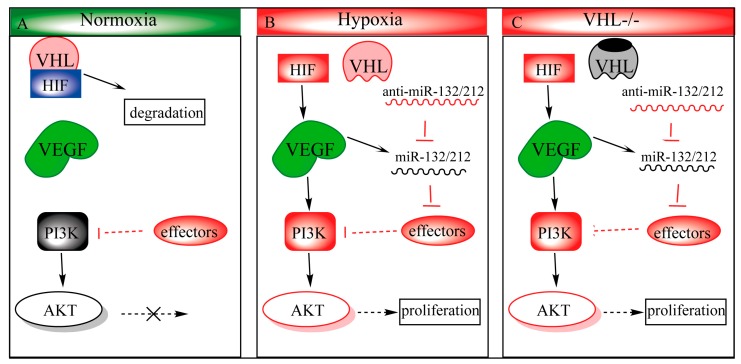
Proposed mechanism of miR-132/212 in modulation of the VHL/phosphatidylinositol-4,5-bisphosphate 3-kinase (PI3K)/ Protein kinase B(AKT)pathways. (**A**) During normoxia, hypoxia-inducible transcription factor 1 (HIF1) is ubiquitinated by the VHL-ubiquinition complex, targeting it for degradation. Some effactors, such as *PTEN*, antagonizes PI3k to prevent AKT from being activated. (**B**) Upon hypoxia, HIF1 can no longer be hydroxylated, which prohibits VHL-regulated degradation, and allows stabilized HIF1 to translocate to the nucleus, upregulating its downstream targets such as *vascular endothelial growth factor* (*VEGF*). VEGF in turn activates the PI3k-AKT pathway and upregulates miR-132/212 expression as well. Upregulated miR-132/212 inhibits effector (e.g., *PTEN)* expression, which in turn prolongs AKT activity. (**C**) *VHL* loss-of-function phenocopies hypoxic conditions even in the presence of oxygen (pseudo-hypoxia).

*vhl*^−/−^ zebrafish embryos display a phenotype of post-vascularization branching/sprouting around the intersomitic vessels in the tails. Counting these sprouts is known as a quantitative measure of angiogenesis in zebrafish [13]. *vhl*^−/−^ zebrafish were injected at a one-cell stage with anti-miRs directed against miR-132 or miR-212, and four days later, tails of the living fish were imaged with a confocal microscope (Figure 3A). The cloaca of the zebrafish is placed in the center of the image, and the branches sprouting from the inter-somitic vessels were counted for the four vessels anterior and the four vessels posterior to the cloaca (Figure 3B). Anti-miR injections against miR-212/132 significantly reduced the extent of intersomitic vessel sprouting in *vhl*^−/−^ fish (Figure 3C,D). In addition, injecting wild-type zebrafish with miR-212/132 mimics partially recapitulated the *vhl* mutant vessel sprouting phenotype (Supplemental Figure S1C,D). In light of the fact that miR-212/132 expression is therefore linked to vessel sprouting in *vhl*^−/−^ zebrafish, we proceeded to look at the expression of PTEN in our VHL-null models, as we had previously done in HUVECs. Zebrafish, as opposed to mammals, have two copies of the *pten* gene: *ptena* and *ptenb.* Zebrafish with loss of both *ptena* and *ptenb* [17] also display a vessel sprouting phenotype that phenocopies the one found in zebrafish injected with miR-212/132 mimics. *ptenb* is a predicted target of miR-212/132 in targetscan but not *ptena* (Supplemental Figure S1E). Accordingly, we found significantly reduced *ptenb* expression in *vhl*^−/−^ zebrafish with no significant changes observed in *ptena* (Figure 4E).

## 4. Discussion

In this study, we used patient material, human cells, and zebrafish to examine the role of the miRNA-212/132 family in ccRCC tumor neovascularization caused by the loss-of-functional pVHL. We observed that miR-212/132 is upregulated in response to *VHL* mutation both in zebrafish model systems and in human patient ccRCC tumor material carrying biallelic inactivating *VHL* mutations. We demonstrated that the excessive angiogenesis attributable to *VHL* mutation is strongly affected by miR-212/132. Indeed, targeting these miRNAs with anti-miRs can significantly reduce angiogenesis in both in vitro and in vivo models of *VHL* deficiency. We identified the tumor suppressor PTEN as one of the targets affected by miRNA-212/132 in *VHL*-null models. Taken together, our results implicate miRNA-212/132 as an important intermediate in angiogenesis after loss-of-functional pVHL or tissue hypoxia.

The miR-212/132 family is clustered in the genome and is highly conserved in vertebrates. miR-212/132 is initially expressed as one primary miRNA and then processed into two mature miRNA with the same target-defining “seed” sequence [18]. This miRNA family plays a number of roles in the promotion of angiogenesis. Mice without functional miR-212/132 show impaired arteriogenesis response after hindlimb ischemia [19]. The pro-angiogenic potential of miR-132 has been used to increase angiogenesis in endothelial cell grafts and after ischemic injury [20]. miR-212/132 frequently act as a promoter of cell proliferation, and increases in their expression levels have also been suggested as contributors to tumorigenesis in addition to their angiogenic role. miR-132 has previously been shown to induce neovascularization in the endothelium by targeting p120 Ras GTPase-activating protein [5]. In addition, anti-miR-132 has also been shown to reduce tumor burden in a mouse xenograft model of human breast carcinoma [5].

This study supports the previously reported role of miR-212/132 in angiogenesis and expands upon its role in the context of VHL-regulated hypoxia signaling. When VHL is mutated or downregulated, miR-212/132 is consequently upregulated. miR-212/132 targeting of mRNA such as *PTEN* ensures that downstream effectors are upregulated. For example, in the case of *PTEN*, PI3K may activate AKT, leading to an increase in proliferation. Indeed, cysts taken from VHL patients display hyperactivation of PI3K signaling [21]. Due to technical reasons, we were not able to detect this increased AKT signaling directly in our zebrafish model. In addition, *Pten* ^−/−^ *Vhl* ^−/−^ double mutant mice develop benign squamous metaplasia and cystadenoma [21] and display kidney cysts that are very similar to those taken from the kidneys of human VHL patients, while mouse models with *Vhl* mutations only do not develop renal tumors [22]. Uncontrolled proliferation and angiogenesis are hallmarks of cancer, and many tumors contain mutations leading to hyperactivation of signaling networks which act to promote these processes [23,24,25]. Differential expression of miRNA has been previously reported in tumors including ccRCC [26] and is widely believed to be an important player in tumorigenesis [27,28,29]. A group of miRs, termed hypoxamiRs, has been shown to be upregulated in hypoxia and play a role in the modulation of cellular responses to a lack of oxygen. The hypoxamiRs include miR-21; 23; 24; 26; 103/107; 373; and, most well studied, miR-210. Hypoxia is also considered a hallmark of the microenvironment of solid tumors, and a number of hypoxamiRs have been implicated in tumorigenesis [30,31]. Thus, the action of miRNA may play an important role in tumorigenesis and therefore presents an interesting potential target for the treatment of cancer.

One of the hallmarks of ccRCC is resistance to cytotoxic treatment. Antiapoptotic signaling is upregulated after HIF1α hyper-stabilization in ccRCC tumors. Many experimental treatments focus on inhibiting the action of downstream antiapoptosis proteins such as mammalian target of rapamycin (mTOR), an important pro-survival protein induced by activated AKT signaling [32]. Our results and the results of other studies suggest that miR-212/132 may act as a promoter of tumorigenesis by targeting inhibitors of proliferation, survival, and angiogenesis, presenting an interesting opportunity for pharmaceutical intervention. Currently, medications which target miRNA are largely unexplored as an avenue by which to target cancer. Here, we have shown that antagonizing the activity of this miRNA family can reduce angiogenesis in relevant models of *VHL* deficiency. *PTEN*, which we have confirmed to be a target of the miR-212/132 family in these models, is frequently mutated in cancer. Another miRNA, miRNA-21, has previously been shown to target PTEN in multiple cancer types and has been implicated in tumorigenesis [33]. Loss of PTEN leads to upregulation of mTOR signaling, and mTOR inhibitors have been used to treat multiple cancer types. Therefore, we envision that treatments designed to antagonize the effect of miRNA-212/132 or other miRNA might be able to reduce the angiogenic burden of tumors in patients, to reduce tumor resistance to other chemotherapeutic agents, or to slow or halt tumor growth.

Conversely, treatments with miR-212/132 might also be a useful method to promote angiogenesis and to increase neovascularization in the cases where neovascularization would be helpful, such as certain ischemic tissues or newly transplanted engineered tissue constructs. In fact, ex vivo transfection of mir-132 into endothelial cells has been shown to be beneficial for transplantation and vascularization of transplanted endothelial cells [34]. Based on our results and the results of other studies, further work is warranted in this area.

## Supplementary Materials

The following are available online at https://www.mdpi.com/2073-4409/9/4/1017/s1, Figure S1: Upreguation of miR-132/212 improve neovascularization in WT Zebrafish.
