# Supplementary material for: Control of Angiogenesis via a VHL/miR-212/132 Axis
Source: Cells. 2020 Apr 19;9(4):1017. doi: 10.3390/cells9041017 (PMC7226144; doi:10.3390/cells9041017)
Supplement: Supplementary file 1 [file cells-09-01017-s001.pdf]

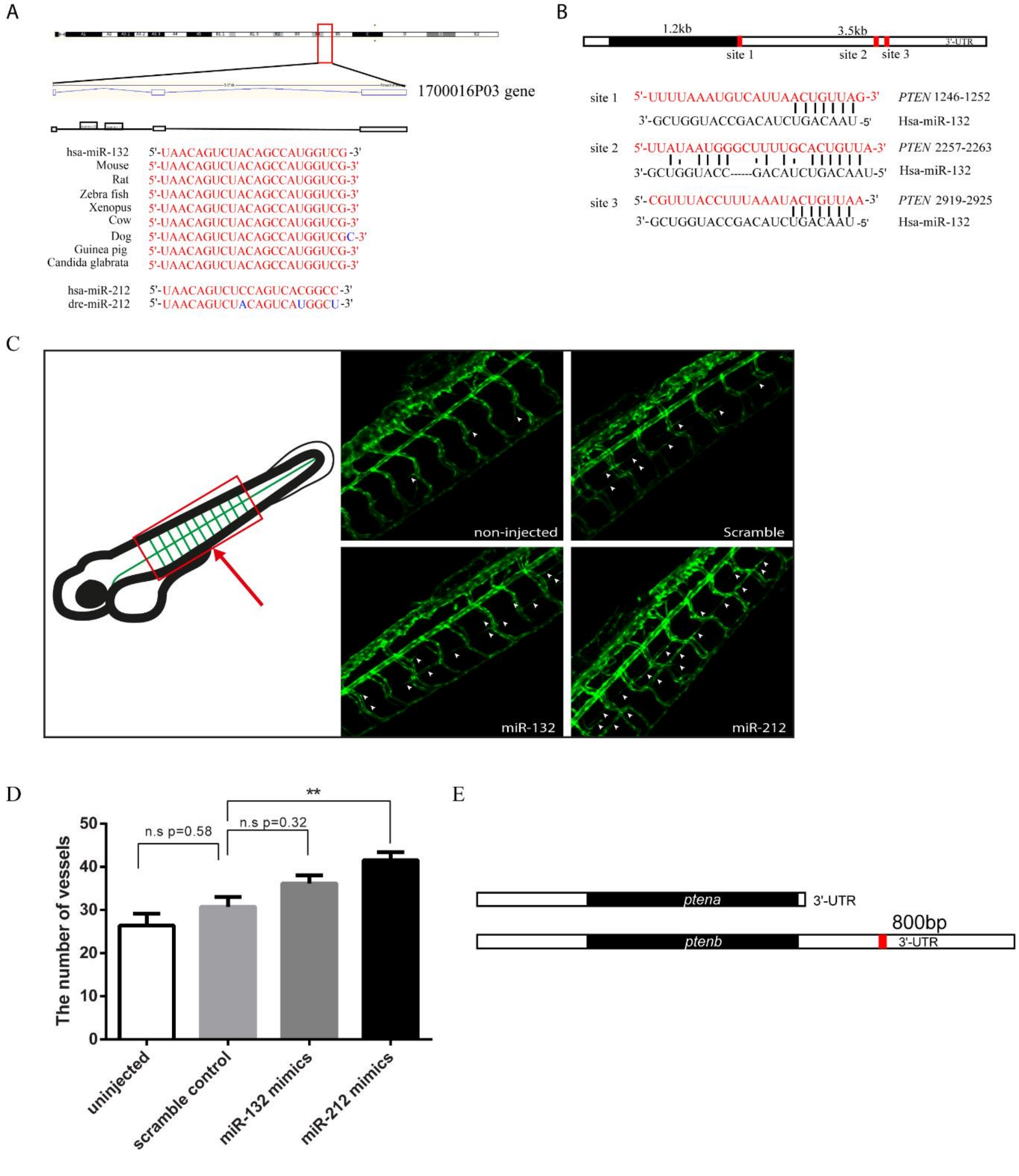

Supplementary Figure S1. Upreguation of miR-132/212 improve neovascularization in WT Zebrafish, A. miR-132/212 location in human genome and conservation of miR-132/212 between different species. B. Three predicted miR-132 target sites on PTEN predicted by Targetscan in human. Target sites and sequences are shown in red. C. Representative images of

tail vascular structures in WT zebrafish after being injected with scrambled, miR-132, or miR-212 mimics. Schematic cartoon shows the area of the zebrafish embryo shown (cloaca marked with red arrow, imaging area marked with red box). D. Quantification of vascular branching after injected with scrambled, miR-132, or miR-212 mimics. E.The position of predicted miR-132 targets on zebrafish orthologues ptena and ptenb by Targetscan. Note that only ptenb has a predicted miR-132 targeting site on the 3'-UTR (red).
